# Characterizing HSF1 Binding and Post-Translational Modifications of *hsp70* Promoter in Cultured Cortical Neurons: Implications in the Heat-Shock Response

**DOI:** 10.1371/journal.pone.0129329

**Published:** 2015-06-08

**Authors:** Andrea V. Gómez, Gonzalo Córdova, Roberto Munita, Guillermo E. Parada, Álvaro P. Barrios, Gonzalo I. Cancino, Alejandra R. Álvarez, María E. Andrés

**Affiliations:** Department of Cellular and Molecular Biology, Faculty of Biological Sciences, Pontificia Universidad Católica de Chile, Av. Libertador Bernardo O’Higgins 340, Santiago, Chile; Boston University Medical School, UNITED STATES

## Abstract

Causes of lower induction of Hsp70 in neurons during heat shock are still a matter of debate. To further inquire into the mechanisms regulating Hsp70 expression in neurons, we studied the activity of Heat Shock Factor 1 (HSF1) and histone posttranslational modifications (PTMs) at the *hsp70* promoter in rat cortical neurons. Heat shock induced a transient and efficient translocation of HSF1 to neuronal nuclei. However, no binding of HSF1 at the *hsp70* promoter was detected while it bound to the *hsp25* promoter in cortical neurons during heat shock. Histone PTMs analysis showed that the *hsp70* promoter harbors lower levels of histone H3 and H4 acetylation in cortical neurons compared to PC12 cells under basal conditions. Transcriptomic profiling data analysis showed a predominant usage of cryptic transcriptional start sites at *hsp70* gene in the rat cerebral cortex, compared with the whole brain. These data support a weaker activation of *hsp70* canonical promoter. Heat shock increased H3Ac at the *hsp70* promoter in PC12 cells, which correlated with increased Hsp70 expression while no modifications occurred at the *hsp70* promoter in cortical neurons. Increased histone H3 acetylation by Trichostatin A led to *hsp70* mRNA and protein induction in cortical neurons. In conclusion, we found that two independent mechanisms maintain a lower induction of Hsp70 in cortical neurons. First, HSF1 fails to bind specifically to the *hsp70* promoter in cortical neurons during heat shock and, second, the *hsp70* promoter is less accessible in neurons compared to non-neuronal cells due to histone deacetylases repression.

## Introduction

Heat, free radicals, bacterial infections, heavy metals, among other stresses, turn on the heat shock response in cells. This program consists of a fast and transitory increase of heat shock proteins (hsp) favoring cells survival [1]. The induction of hsp genes is regulated by the transcription factor Heat Shock Factor 1 (HSF1). Under basal conditions, HSF1 rests in the cells as an inactive monomer. Stressful stimuli induce HSF1 trimerization and its nuclear permanency. HSF1 binds the Heat Shock Element (HSE) present in the promoter of hsp genes, where it is finally activated by phosphorylation allowing competence for transcriptional activation [2].

Hsp70 is one of the most conserved proteins in nature [3] characterized by being one of the most highly induced in response to stress [4]. Even though the stress response is a general conserved cellular program, different cell populations present differential capacity to induce Hsp70 expression during stress. Neuronal cells do not induce or induce lower levels of Hsp70 in response to stressful stimuli [5–10]. Moreover, neuronal differentiation programs decrease heat shock response. For instance, PC12 differentiation to pseudo sympathetic neuronal phenotype by neuronal growth factor (NGF) treatment [11] decreases their capacity to induce Hsp70 in response to heat shock and ethanol treatments [12,13]. The lower capacity of neurons to induce Hsp70 during stress may have important implications for the vulnerability to neurodegenerative diseases [14–16]. For, instance, overexpression of Hsp70 reduces neuronal dystrophy in a mouse model of Parkinson’s disease [17] and Hsp70 reduction by miR-61-1 increases α-synuclein aggregation in SH-SY5Y cells [18].

Several mechanisms have been studied to clarify why neurons display lower induction of Hsp70 in stress. Marcuccilli et al. [19] suggested a more important role for HSF2 since HSF1 was barely detected in neurons. It has been shown that even though HSF1 is present in neurons, there is a lack of proper activation during heat shock [20]. Other scientists have suggested a negative role for chromatin on *hsp70* gene expression in neuronal cells, thus preventing the access of HSF1 and other transcription factors to the promoter [7,10]. Moreover, it was shown that HSF1 does not bind DNA under stress in cell lines with neuronal phenotype [7,21]. This proposal has been reinforced by data showing that histone deacetylase (HDAC) inhibitors increase hsp70 transcription in neurons [22,23]. Additionally, Guertin and Lis [24] showed in *Drosophila* by genome-wide analysis that an active chromatin landscape around HSEs is required for HSF1 binding elicited by heat shock. These data indicate that the regulation of Hsp70 expression and neuroprotection mechanisms during stress in neurons are still poorly understood.

Post-translational modifications (PTMs) of the N-terminal tail of histones underlie chromatin status regulating gene expression. Acetylation of histones marks actively transcribed chromatin while closed chromatin is characterized by unacetylated histones. On the other hand, methylation on specific residues distinguishes actively transcribed genes from repressed ones. Di- and tri-methylated lysine 4 on histone H3 (H3K4me2, H3K4me3) and unmethylated K9 on histone 3 (H3K9me0) are features of actively transcribed genes, and the opposite marks are found in repressed genes [25]. The importance of PTMs of histones on *hsp70* promoter in response to stress has been shown in yeast and *Drosophila* and increasing data is available in mammalian genomes [26].

In this work, rat transcriptomic databases and cultured rat cortical neurons were used to study HSF1 expression, nuclear translocation and binding to DNA. In addition, chromatin PTMs at the *hsp70* promoter, in basal and stress conditions, were analyzed to inquire further into the mechanisms that decrease stress-dependent induction of Hsp70 in neurons. Altogether, the data show that cortical neurons display lower response to heat shock even though HSF1 is present and activated. Strong HDAC-dependent repression and a specific failure of HSF1 binding to the *hsp70* promoter weakens Hsp70 induction in cortical neurons.

## Materials and Methods

### Primary Culture of Cortical Neurons

Primary cultures of rat cortical neurons were prepared from E18 embryos, obtained from timed pregnant Sprague-Dawley rats (Animal Care Facility of the Faculty of Biological Sciences, Pontificia Universidad Catolica de Chile) decapitated using a guillotine. Every effort was made to reduce the chance of pain or suffering. The procedures were approved by the Bioethical Committee of the Faculty of Biological Sciences of the Pontificia Universidad Católica de Chile and were performed in strict accordance with the guidelines published in ‘‘NIH Guide for the Care and Use of Laboratory Animals” and “Guidelines for the Use of Animals in Neuroscience Research” by the Society for Neuroscience.

Cortical cells prepared as described [27] were plated on poly-L-lysine-coated wells and maintained in Neurobasal medium supplemented with B27, 100 U/ml penicillin and 100 μg/ml streptomycin for 7 days in vitro (DIV), before any experimental manipulation. To inhibit glial proliferation, 2 μM Cytosine-Arabinoside [28] was added on the second day of culture and removed by changing the medium 24h later. Unless otherwise indicated, all cell culture reagents were acquired from Invitrogen Corporation.

### PC12 differentiation protocol

PC12 cells (ATCC) were cultured in Dulbecco’s modified Eagle’s medium (DMEM) supplemented with 10% horse serum, 5% fetal bovine serum, 1% penicillin/streptomycin and maintained at 37°C, 10% CO_2_. For neuronal differentiation experiments, PC12 cells were plated at 5x10^3^ cells/cm^2^. Twenty four hours later, the medium was replaced by DMEM supplemented with 2% horse serum plus 50 ng/ml of NGF (Alomone Labs, Ltd). Cells were maintained for 7 days, changing the media with fresh NGF every two days, prior to heat shock treatment. Undifferentiated PC12 cells were cultured under low serum conditions but without NGF.

### Heat Shock and Drug Treatments

Heat shock was induced by incubating PC12 and cortical neuron cultures for 2h in a preheated water bath at 42°C. Any modification to this heat shock protocol is indicated in figure legends. Trichostatin A (TSA; Sigma-Aldrich) was directly added to the culture medium at the desired concentration ranging from 2.5–40 nM. Cells were incubated with the drug for 15h and harvested afterward.

### Preparation of Nuclear Fractions and Western Blotting

PC12 cells and cortical neurons were washed three times and scrapped in ice-cold phosphate-buffered saline (PBS). Whole-cell extracts were obtained by resuspending the cells with 1ml syringe in Triton X-100 lysis buffer (50mM Tris-HCl pH 7.5, 1% Triton X-100, 150mM NaCl, 1mM PMSF plus protease inhibitors) and left on ice for 20min. Homogenates were centrifuged at 14000 rpm for 20 min at 4°C and supernatants were saved for further analysis. Samples were resolved on SDS-PAGE and proteins detected by western blot with a mouse monoclonal antibody against Hsp70 (Stressmarq) and a rabbit polyclonal antibody against HSF1 (Cell Signaling Technology, Inc). Quantification of Hsp70 induction was carried out as described before [29] using α-tubulin (mouse monoclonal antibody; Sigma) and GAPDH (mouse monoclonal antibody; Zymed, Millipore) as loading controls. The nuclear fraction from cortical neurons was obtained as described previously [30] with one modification. After the wash of the nuclear fraction, the pellet was resuspended in Triton X-100 lysis buffer as described above, and 25μg of nuclear protein was used for western blotting.

### Quantitative Real-Time PCR

Total RNA was extracted from cells using TRIzol reagent (Invitrogen Corporation) following manufacturer’s instructions; 500ng of RNA were subjected to reverse transcription using MMLV-RT (Fermentas International Inc). Quantitative Real-Time PCR analysis was performed using a LightCycler (Roche Applied Science) as described before [31]. Cyclophilin A (CYC) was used as the internal reference gene. The following primers were used to amplify target cDNAs: Hsp70c-F (5’-AACTACAAGGGCGAGAACCGGTC-3’)

Hsp70c-R (5’-GATGATCCGCAGCACGTTCAGA-3’)

Hsp25c-F (5’- ACTCAGCAGCGGTGTCTCAGAGATCC-3’)

Hsp25c-R (5’-GGTGAAGCACCGAGAGATGTAGCCA-3’)

CYC-F: (5’-TGCTCTGAGCACTGGGGAGAAA-3’)

CYC-R: (5’-CATGCCTTCTTTCACCTTCCCAAAGAC-3’).

### Plasmids

Myc/His-hHSF1 expression vector encoding full-length human HSF1 was previously described [29,32]. Hsp70B-luc reporter plasmid was generated by subcloning a BglII-HindIII fragment (1.44kb) containing human Hsp70B gene promoter from p2500CAT vector (Stressgen Biotechnologies Corp; [33]) into pGL3-basic vector (Promega).

### Reporter Gene Assays

Transient transfection and reporter gene assays were performed as previously described [34]. Briefly, PC12 cells (1.5x10^5^ cells/well; 24 wells plate) were transfected with 515ng of total DNA by using Lipofectamine 2000 reagent (Invitrogen). Hsp70B-luc reporter plasmid (149.3ng) was used in 1:1 molar ratio respect to Myc/His-hHSF1 expression plasmid. DNA was kept constant by adding pBLUescript (Stratagene), and in every experiment 25ng of the pCMX-β-gal reporter vector was cotransfected as control of transfection efficiency.

### Chromatin Immunoprecipitation Assay

ChIP assays from PC12 cells (7x10^6^ cells) and cortical neurons (1x10^7^ cells) were performed as previously described [29]. Immunoprecipitations were carried out with the following rabbit polyclonal antibodies: anti-H3Ac, anti-H4Ac, anti-H3K4me2, H3K9me2 (Upstate, Millipore); anti-H3K4me3, anti-H3 (Abcam) and anti-HSF1 (Santa Cruz, Biotech). Rabbit Pre-immune IgG (Santa Cruz, Biotech) and no antibody were used as a control of immunoprecipitation specificity. Quantitative real-time PCR (qPCR) analysis was performed using a LightCycler (Roche Applied Science). One μl of each sample was subjected to qPCR using the following primers: rHsp70pr-F (5’-ACACTTGTCACAACCGGAACAAGC-3’), rHsp70pr-R (5’-TCTCTGCGAGTGGAACCAGAAACT-3’), rHsp25-F1 (5’-GACAGTGGGAACTGCTCCAG-3') and rHsp25-R1: (5'-TGGCAATGACCGTCTAAGGG-3'). Standard curves were generated by using serial dilutions of previously quantified genomic DNA. The amount of immunoprecipitated DNA in each sample was calculated using fit point analysis with no baseline adjustment. Data was normalized to the Percentage of the Input Method for further analysis. When comparing histone PTMs of the *hsp70* (*hspa1b)* or the *hsp25* (*hspb1*) rat gene promoters between cortical neurons and PC12 cells, ChIP signals were normalized relative to nucleosome density. In this case, the ChIP-qPCR signals obtained with every antibody were divided by the signal obtained with anti-H3 [35].

### Bioinformatic Analysis

RNA-seq data of rat whole brain (SRR594428, male breeding age) and cerebral cortex (SRR388230, SRR388232 and SRR388234, adult male) were used. Whole brain data were sequenced by Illumina HiSeq 2000 [36], and the reads were processed to remove the adapters and low-quality sequences with FASTX toolkit (http://hannonlab.cshl.edu/fastx_toolkit/index.html). The resultant reads were aligned to the rat reference genome (rn5) using TopHat [37]. Cerebral cortex data were sequenced by AB SOLiD 4 System using a strand-specific library preparation protocol [38]. The 50 nt reads were trimmed to 36 nt. The resultant reads were aligned using Bowtie [39] to the rat reference genome (rn5). We used CAGE data from rat brain (8–12 weeks) and cerebral cortex (DRP000155, 3–28 days) [40] and PolyA-seq data from rat brain (SRX080235) [41]. The reads were processed to remove the adapters and low-quality sequences and were mapped to the rat reference genome (rn5) using Bowtie. All the alignments were visualized as the UCSC Genome Browser tracks [42]. An integration of RNA-seq, CAGE-seq, and PolyA-seq data were used to propose a more accurate annotation of Hsp70.

### Statistical analysis

Results are presented as mean ± SEM from at least three independent experiments. The results were analyzed for statistical significance as described in each figure legend, using GraphPad PRISM version 6.0.

## Results

### Neuronal cells show reduced induction of Hsp70 in response to heat shock

**Fig 1:**
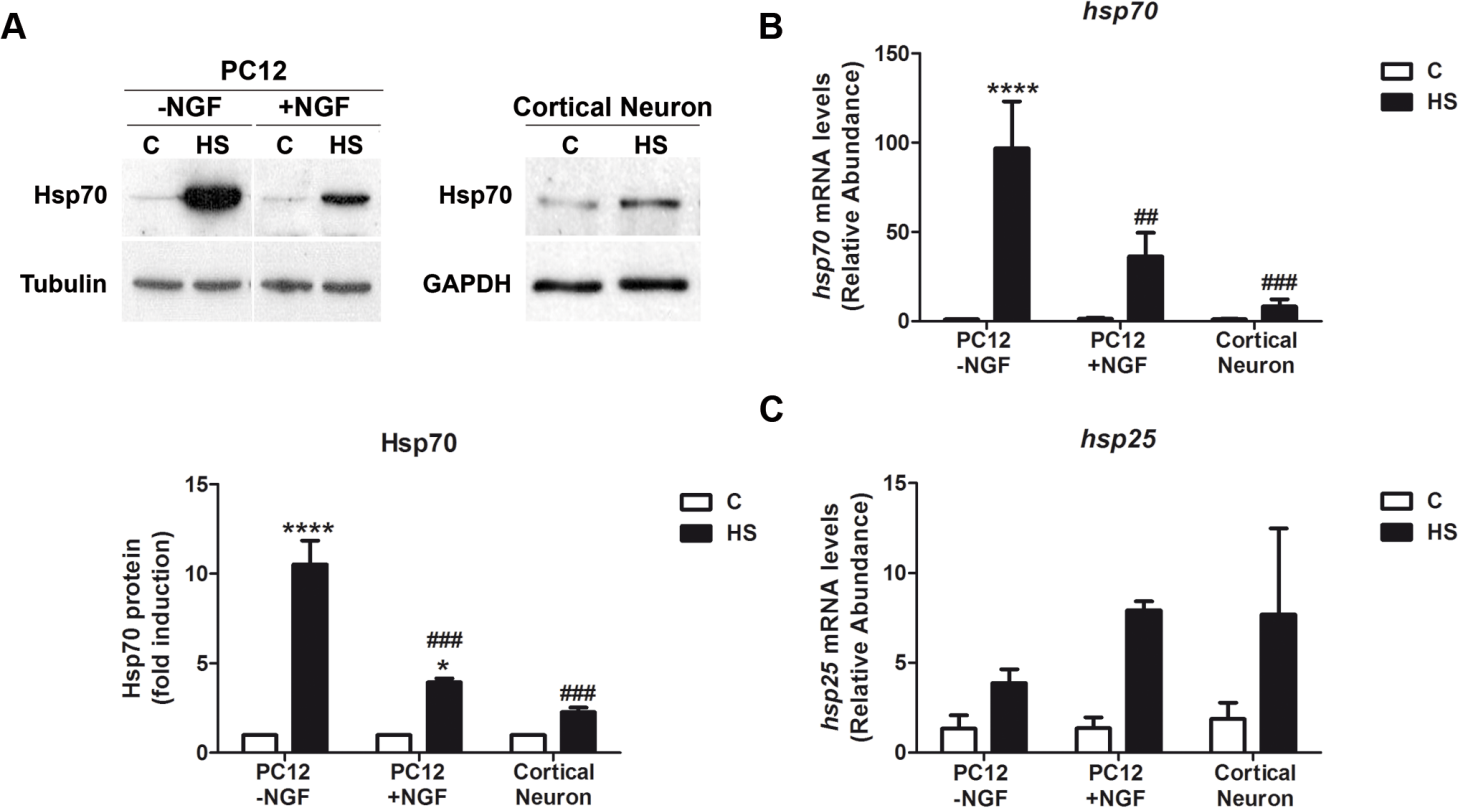
Neuronal cells show weaker induction of Hsp70 in response to heat shock. (A) Upper panels: Representative immunoblots of Hsp70 protein in undifferentiated (-NGF) and differentiated (+NGF for 7d) PC12 cells, and cortical neurons (E18.5, 7div) subjected to heat shock (42°C, 2h) or kept under control conditions (C, 37°C). Lower panel: Quantification of the relative Hsp70 protein levels showed in the upper panels. Tubulin and GAPDH were used as loading controls. Statistical analyses were performed by Two-way ANOVA followed by Tukey’s multiple comparisons test. ****p < 0.0001, *p < 0.05 compared with control; ###p <0.001, compared to Hsp70 fold induction in undifferentiated PC12 cells during heat shock. (B, C) Relative abundance of hsp70 and hsp25 transcripts in PC12 cells and cortical neurons. hsp70 and hsp25 mRNA levels were calculated comparing the abundance of each cDNA in cells under control and heat shock conditions. For each sample, cyclophilin mRNA was used as a reference gene. Data are expressed as mean plus SEM of at least three independent experiments. Statistical analyses were performed by Two-way ANOVA followed by Tukey's multiple comparisons test. ****p < 0.0001, *p < 0.05 compared with control; ##p < 0.01, ###p <0.001, compared to Hsp70 mRNA abundance in undifferentiated PC12 cells during heat shock.

Primary cultures of rat cortical neurons, and undifferentiated and NGF-treated PC12 cells were exposed to 42°C for 2h. Hsp70 protein levels are low under basal conditions in every cell type analyzed. The amount of Hsp70 protein increased in the three cell types in response to heat shock, even though the data confirm that the increment of Hsp70 in cells with neuronal phenotype, such as NGF-treated PC12 cells (PC12 plus NGF, 3.9 ± 0.3 fold of induction) and cortical neurons (2.4 ± 0.6 fold of induction) is significantly lower than in non-neuronal cells (undifferentiated PC12 cells; 8.2 ± 0.2 fold of induction) (Fig 1A). This lower induction of Hsp70 by heat shock in neurons is also observed at transcriptional level. After 2h of heat shock, the mRNA of hsp70 is induced 96.8 times in undifferentiated PC12 cells while in cortical neurons is induced only 8.9 times (Fig 1B). This effect is specific for *hsp70* gene since hsp25 mRNA is induced to a similar extent in cortical neurons and, NGF-treated and *naïve* PC12 cells (Fig 1C). These data suggest that the neuronal differentiation process decreases the capability of inducing *hsp70* gene transcription by heat stress.

### Transcriptomic profiling data of whole brain and cerebral cortex show a differential transcriptional start sites usage of *hsp70* gene

**Fig 2:**
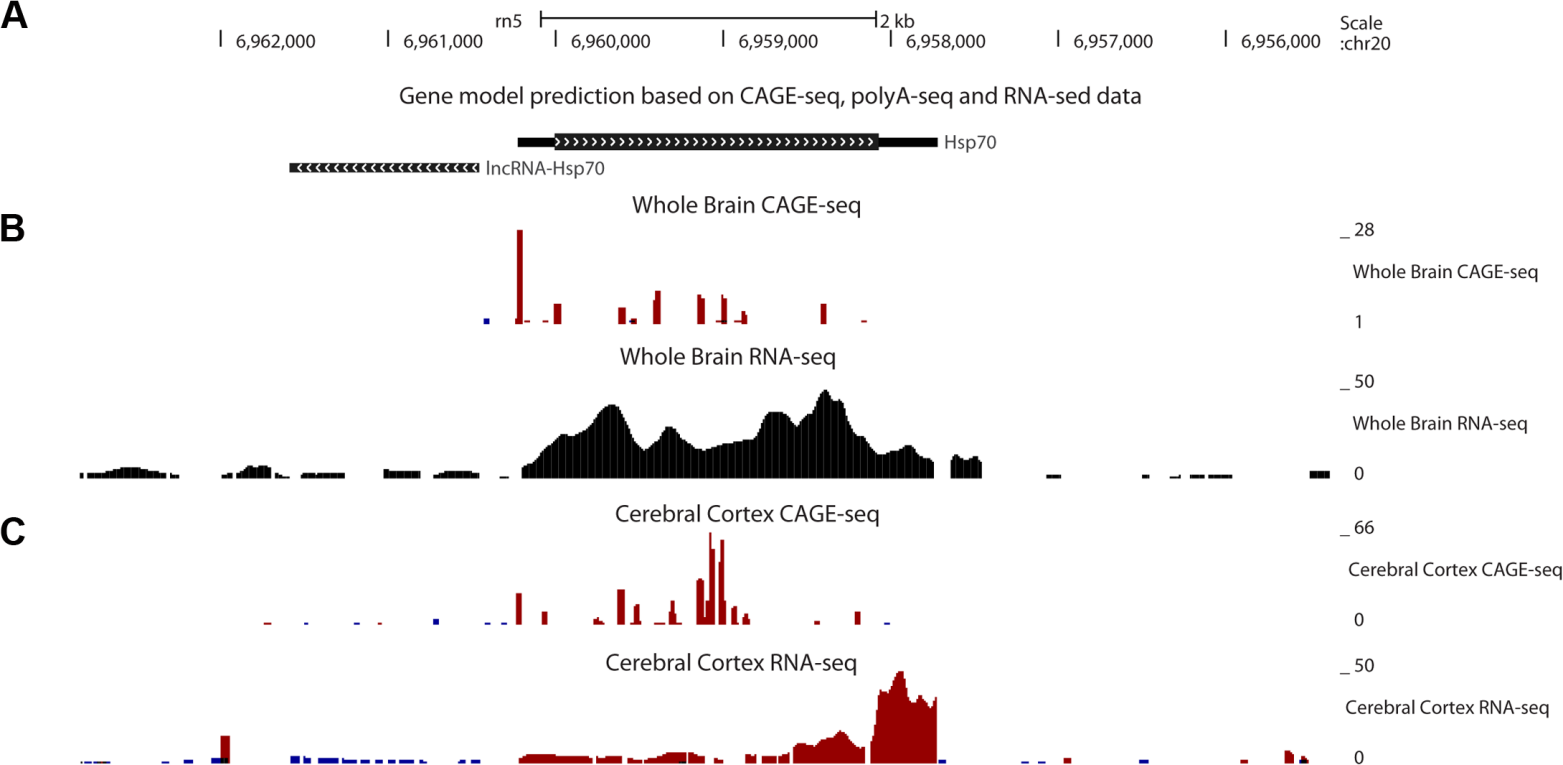
The *hsp70* gene shows predominant cryptic transcription initiation sites usage in rat cerebral cortex. (A) Model of rat *hsp70* gene based on our transcriptome profiling data analysis. (B) Whole brain transcriptome profiling data shows a predominant transcription start site at the beginning of the gene. (C) Cerebral cortex transcriptome profiling data shows predominant transcriptional start sites inside the codifying sequence of *hsp70 gene*. The coverage for reads aligned in the positive and negative strand are shown in red and blue, respectively.

To look for *in vivo* data of hsp70 transcriptional pattern, we studied the transcriptomic profile of rat *hsp70* gene in the cerebral cortex and whole brain. Based on RNA-seq, CAGE-seq and PolyA-seq data, we annotated a rat *hsp70* gene model (Fig 2A). Transcriptome profiling data show a predominant usage of the bidirectional promoter of *hsp70* in the whole brain but not in the cerebral cortex. In cerebral cortex the highest CAGE-seq peaks are located inside the *hsp70* open-reading frame (ORF), which is coincident with a RNA-seq coverage increase at the 3’ end of hsp70 (Fig 2B and 2C), indicating that differential transcriptional start sites usage occurs in cortical cells. This data suggests that cryptic promoters are activated inside *hsp70* ORF in cortical cells, and this is due to a weaker activation of the canonical promoter of *hsp70*.

### Heat shock induces HSF1 nuclear translocation; although HSF1 does not bind to *hsp70* promoter in cortical neurons

**Fig 3:**
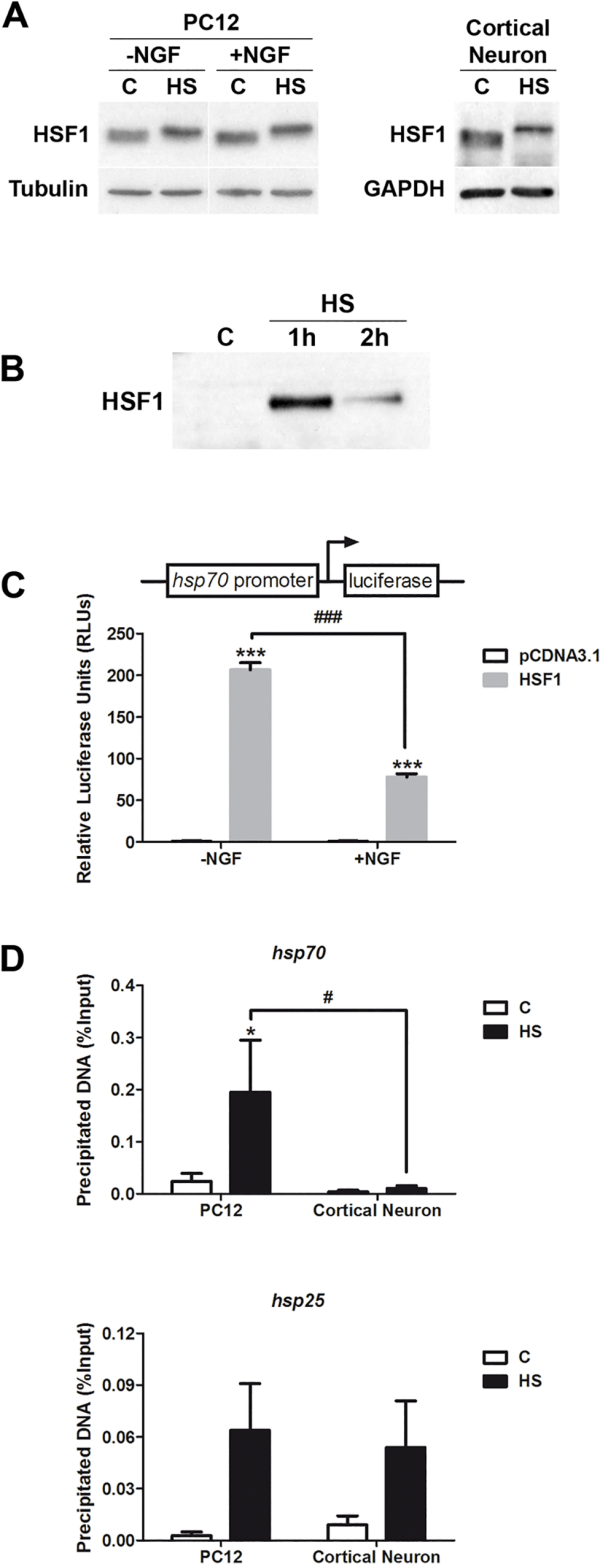
HSF1 does not bind to the *hsp70* promoter in neurons during heat shock. (A) Representative immunoblots of HSF1 protein expression in undifferentiated (-NGF) and differentiated (+NGF for 7d) PC12 cells, and in cortical neurons (E18.5, 7div) subjected to heat shock (42°C, 2h) or kept at control conditions (C, 37°C). (B) Nuclear fractions from cortical neurons at control or, after 1h or 2h of heat shock were analyzed for HSF1 protein detection. (C) HSF1 ability to activate *hsp70* promoter in PC12 cells was assayed after 24 h of NGF (50 ng/ml) treatment. Data correspond to the mean plus SEM of three independent experiments. Statistical analyses were performed by Two-way ANOVA followed by Tukey's multiple comparisons test: ***p <0.001, compared to pCDNA3.1; ###p< 0.001 (D) PC12 cells and cortical neurons were heat shocked at 42°C for 1h or kept unstressed. Chromatin was immunoprecipitated with an anti-HSF1 antibody and amplified by quantitative real-time PCR using primers flanking the promoter area of both *hsp70* (upper panel) and *hsp25* (lower panel) genes. Data are expressed as the percentage of the immunoprecipitated DNA in relation to the Input and correspond to the mean plus SEM of at least three independent experiments. Statistical analyses were performed by Two-way ANOVA followed by Tukey's multiple comparisons test: *p < 0.05 compared with control; #p < 0.05 compared to heat shocked PC12 cells.

HSF1 is the main transcription factor responsible for inducing Hsp70 expression during the heat shock response. Thus, a lower induction of Hsp70 in neurons could be due to a lower expression of HSF1 or a failure of its activation in response to stress. Cortical neurons and undifferentiated and NGF-treated PC12 cells were exposed to heat shock to evaluate the following aspects of HSF1: protein levels, transcriptional activity, heat shock-induced nuclear localization and DNA binding ability to the endogenous *hsp70* gene promoter. Western blot assays, performed with an anti-HSF1 antibody, which recognizes both the hypo- and hyper-phosphorylated forms, showed that cortical neurons, and undifferentiated and NGF-treated PC12 cells displayed equivalent amounts of both faster and heat shock-induced slower HSF1 bands (Fig 3A) indicating that HSF1 level is not the limiting factor inducing Hsp70 during heat shock in neuronal cells. Supporting the activation of HSF1 in cortical neurons, one hour after heat shock a strong signal of HSF1 was observed in the nuclear fraction (Fig 3B), showing that HSF1 is efficiently translocated to cell nuclei in response to stress. These results indicate that the initial steps required for HSF1 activation are operative in neurons. To test the transactivation ability of HSF1 in neuronal cells, gene reporter assays were performed using 1.44 Kb of the *hsp70* gene promoter. The graph in Fig 3C shows that following a short stimulation with NGF (24 hours), HSF1 lost 50% of its transactivation ability, confirming that neuronal differentiation process inhibits Hsp70 induction by this transcription factor. The lower reporter activity in NGF-treated PC12 cells could be due to either a failure of the transcriptional activity of HSF1 or a failure of its DNA binding ability. ChIP assays were carried out to compare HSF1 binding to *hsp70* and *hsp25* promoters in cortical neurons and PC12 cells under basal and heat shock conditions. There was no detectable interaction of HSF1 on the *hsp70* gene promoter in cortical neurons under basal or after one hour of heat shock (Fig 3D, upper panel). By contrast, heat shock induced a significant HSF1 binding to the *hsp70* promoter in undifferentiated PC12 cells (Fig 3D, upper panel). Remarkably, HSF1 bound efficiently to the *hsp25* promoter under heat shock conditions in both PC12 and cortical neurons during heat shock response (Fig 3D lower panel). Altogether, the data indicate that the mechanisms activating HSF1 in neurons work properly during heat shock. Moreover, the data suggest that neuronal differentiation may induce changes on chromatin landscape at the *hsp70* gene promoter, preventing HSF1 binding specifically to this promoter in neurons.

### Histone PTM features on *hsp70* gene promoter in cortical neurons and PC12 cells

**Fig 4:**
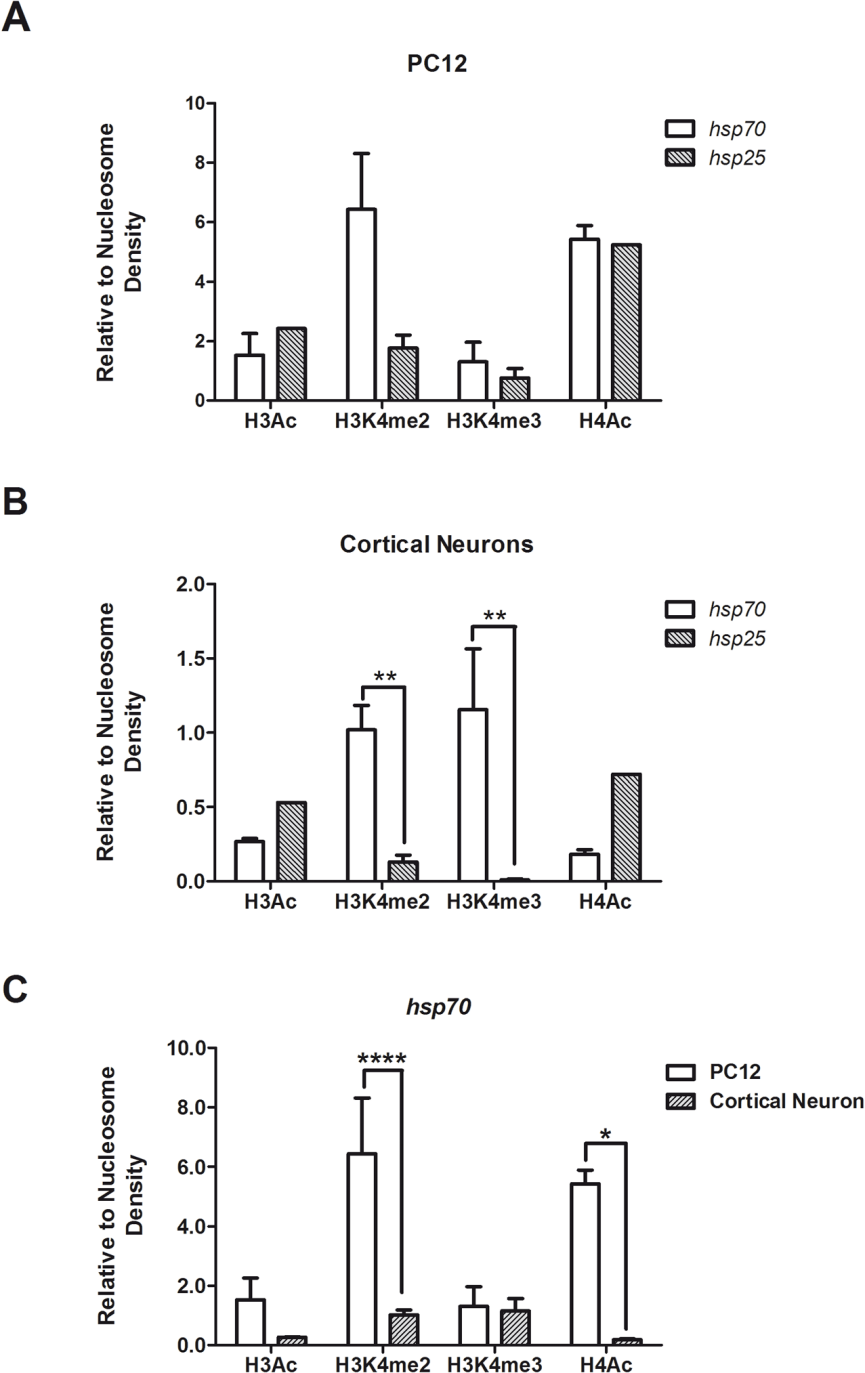
Analysis of the chromatin environment of rat promoter regions of *hsp70* and *hsp25* genes. H3Ac, H4Ac, H3K4me2 and H3K4me3 were analyzed in the proximal promoter region of the *hsp70* and *hsp25* rat genes in undifferentiated PC12 cells (A) and cortical neurons (B), under basal conditions. The graphs show the data from the ChIP assays and correspond to the mean plus SEM of the percentage of DNA immunoprecipitated by each antibody (% of the Input), normalized by the percentage of DNA immunoprecipated by anti-H3 antibody (Relative to Nucleosome Density). Statistical analyses were performed by Two-way ANOVA followed by Bonferroni post hoc multiple comparison test. **p < 0.01. (C) Comparative analysis of the *hsp70* gene promoter between PC12 and cortical neurons. The graph shows the data as described above for (A) and (B). Statistical analyses were performed by Two-way ANOVA followed by Sidak's multiple comparison test. ****p < 0.0001; *p < 0.05.

Transcriptional activators such as HSF1 recognize and bind specific DNA sequence elements depending on chromatin context [43]. To assess whether HSEs on the *hsp70* gene promoter are in a permissive or repressed chromatin context in neurons, ChIP experiments were performed to evaluate H3Ac (K9, K14), H4Ac (K5, K8, K12, K16), H3K4me2, H3K4me3 and H3K9me2 levels. In addition, histone PTMs of *hsp25* promoter in PC12 cells (Fig 4A) and cortical neurons (Fig 4B) were compared. ChIP data show that at basal conditions in PC12 cells, histone PTMs of *hsp70* promoter were similar to those of *hsp25* promoter, except for H3K4me2 that was 3.6 times higher for *hsp70* (Fig 4A). H3K4me2 defines Transcription Factors Binding Regions (TFBRs) [44], associating this feature of *hsp70* promoter with higher binding of HSF1. Surprisingly, higher levels of H3K4me2 were also observed in the *hsp70* promoter in cortical neurons (Fig 4B), while no binding of HSF1 occurred (Fig 3D). Thus, *hsp70* promoter maintains its accesibility to other transcription factors in neurons. Moreover, a higher level of H3K4me3 was also observed in *hsp70* gene promoter in cortical neurons compared with *hsp25* (Fig 4B). H3K9me2, which is associated with transcriptional repression [43], was not detected in the promoter of *hsp70* in any cell type (data not shown). Regarding histone acetylation, *hsp70* promoter acetylation was significantly lower in cortical neurons compared to PC12 cells (Fig 4C). Furthermore, levels of H3Ac and H4Ac in the *hsp25* promoter were higher than in the *hsp70* promoter in cortical neurons (Fig 4B). Altogether, the data suggest that the *hsp70* promoter is more closed in cortical neurons than in non-neuronal cells, even though it presents features of an active promoter.

**Fig 5:**
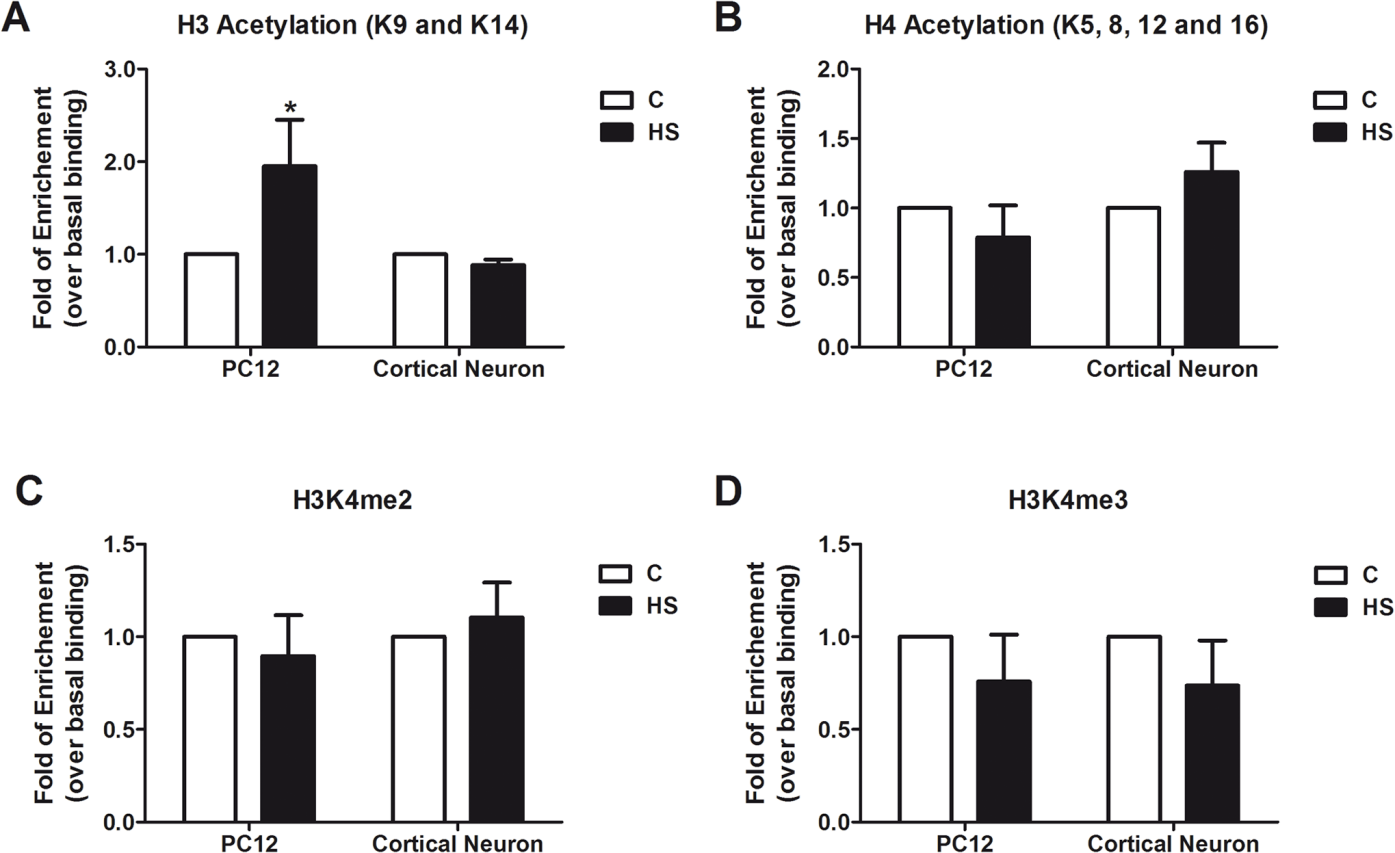
Effect of heat shock on histone PTMs of the rat *hsp70* promoter in PC12 cells and cortical neurons. Comparison of four histone PTM levels: H3Ac (A) and H4Ac (B), H3K4me2 (C) and H3K4me3 (D), at the *hsp70* gene promoter under control (white bars) and heat shock (black bars) conditions, in cortical neurons and PC12 cells. Cells were heat shocked at 42°C for 1h or kept unstressed. Data is expressed as fold of enrichment over basal condition (unstressed) and correspond to the mean plus SEM of three independent experiments. Statistical analyses were performed by Two-way ANOVA followed by Sidak's multiple comparisons test: *p < 0.05 compared to control.

To assess whether heat shock induces changes on histone PTMs that may account for the different degree of Hsp70 induction between neuronal and non-neuronal cells, histone PTMs on *hsp70* promoter during heat shock in cortical neurons and PC12 cells were evaluated. ChIP results showed that heat shock significantly increased H3Ac levels on the *hsp70* promoter in PC12 cells, but not in neurons (Fig 5A). Levels of H4Ac, H3K4me2 and H3K4me3, remained equal during heat shock in cortical neurons and PC12 cells (Fig 5B–5D).

**Fig 6:**
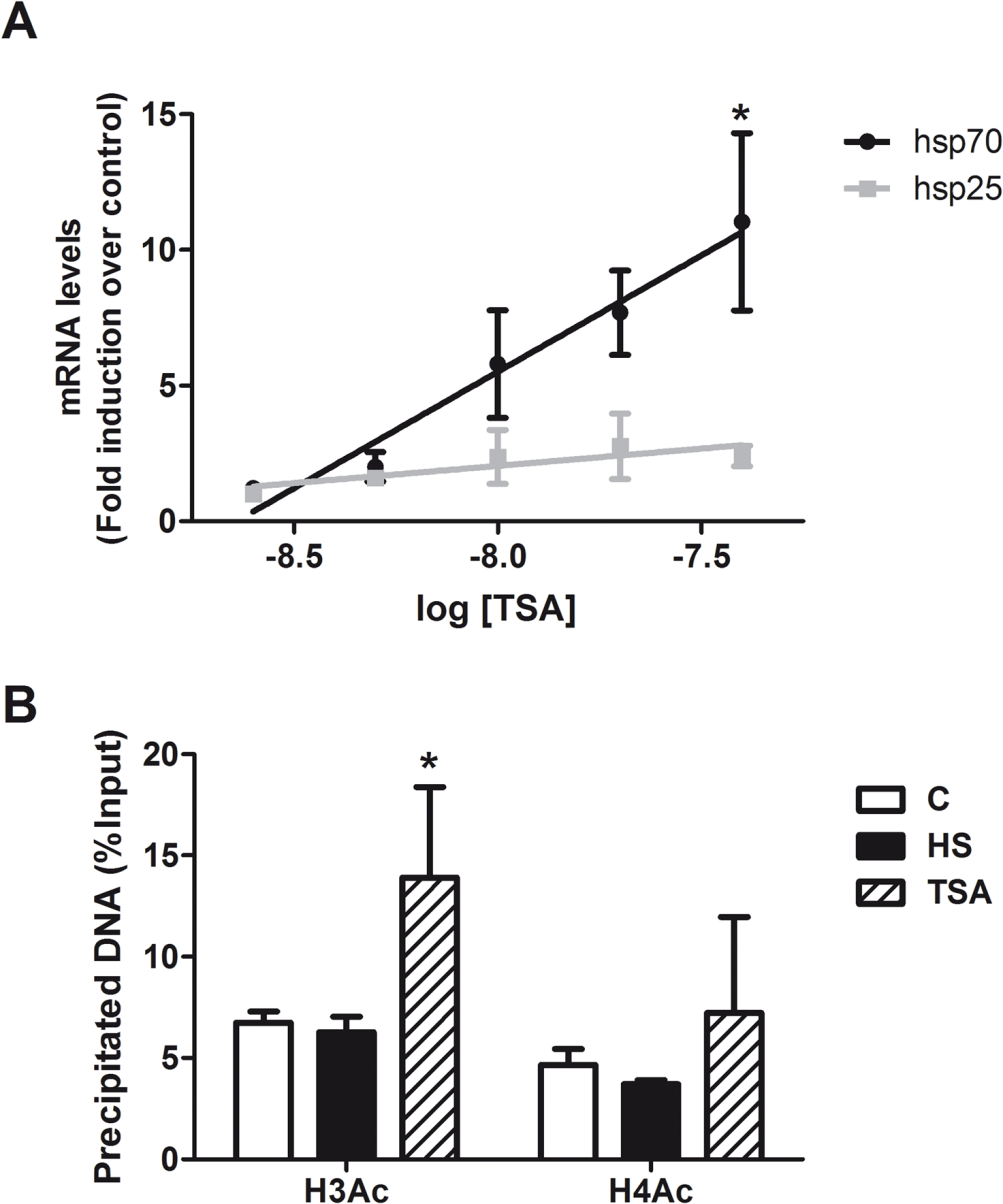
Augmented H3Ac mediated by HDACs inhibition is associated with increased expression of Hsp70 in cortical neurons. (A) Fold Induction of hsp70 (black circles) and hsp25 (grey squares) transcripts in cortical neurons by different doses of TSA. Data are expressed as mean plus SEM of at least three independent experiments. Statistical analyses were performed by best fit Linear Regression and Spearman’s r correlation analysis where *p < 0.05 for hsp70 mRNA. (B) Comparison of histone acetylation of H3 and H4 at the *hsp70* gene promoter in cortical neurons under control, heat shock (42°C, 1h) or TSA (50 nM, 15h) treatment. Data is expressed as the percentage of the immunoprecipitated DNA in relation to the input and correspond to the mean plus SEM of at least three independent experiments. Statistical analyses were performed by Two-way ANOVA followed by Tukey's multiple comparisons test: *p < 0.05 compared to control.

Considering the previous results indicating an association between histone acetylation and Hsp70 induction, we studied the effect of increasing histone acetylation by HDAC inhibition with Trichostatin A (TSA) on hsp70 and hsp25 induction. TSA treatment specifically augmented hsp70 transcript in a dose-dependent manner, while it did not change hsp25 mRNA expression (Fig 6A). Similar results were observed for Hsp70 protein that reached 4.9 times higher levels at 40 nM TSA, compared to the control condition (not shown). The hsp70 expression induced by TSA correlated with a significant increase of H3Ac level at *hsp70* promoter (Fig 5B). TSA treatment did not alter the methylation status of H3K4 (data not shown). These results suggest a direct relationship between H3Ac levels and Hsp70 induction. No HSF1 was detected at the *hsp70* gene promoter in cortical neurons treated with TSA (data not shown), indicating that TSA-induction of Hsp70 in neurons is independent of HSF1 binding to the *hsp70* promoter.

## Discussion

For long time, the decreased stress-dependent induction of Hsp70 in neurons has been subject of debate, with most of the hypotheses pointing to a failure on HSF1 expression or activation [19,20,45,46]. In this report, the evidence indicates that the decreased induction of Hsp70 is related to the failure of HSF1 binding specifically to the *hsp70* promoter in neurons. This data supports a role for changes in chromatin context during neuronal differentiation that hamper HSF1 binding to this gene. The data also suggest a role for histone PTMs on the *hsp70* promoter in this phenomenon. In particular, reduced levels of H4Ac were associated with absence of HSF1 binding to the *hsp70* promoter in neurons.

The results allow discarding a lower abundance of HSF1 as the limiting factor explaining the lower induction of Hsp70 in neurons during heat shock stress. As shown by Western blots, HSF1 is detected in equivalent amounts in undifferentiated and NGF-differentiated PC12 cells, as well as in cultured cortical neurons. Furthermore, heat shock induced HSF1 translocation to the nuclei of neurons, suggesting that HSF1 activation is operative in neurons. However, heat shock failed inducing HSF1 binding at the *hsp70* promoter in neurons, as was showed by ChIP assays, and as it was previously reported in cell lines with neuronal phenotype [7,21].

The analysis of the chromatin landscape showed that the promoter of *hsp70* does not have epigenetic marks for repression in cortical neurons, but present a profile of PTMs indicative of a more closed promoter compared to PC12 cells. Indeed, the results showed that the *hsp70* promoter in neurons exhibited higher levels of H3K4m2 and H3K4me3, compared with *hsp25*, indicative of sites prone for binding of transcription factors and transcriptional activation [25]. However, significant lower histone H4Ac levels were observed in the *hsp70* promoter in neurons compared to non-neuronal cells. Moreover, levels of acetylation of H3 and H4 in *hsp70* promoter are lower than in *hsp25* promoter in cortical neurons. Bioinformatic data also indicated that the *hsp70* promoter is weaker in cortex compared to the whole brain. It has been shown that transcription elongation factors repress transcription initiation from cryptic sites [47–49]. Therefore, a decreased hsp70 transcription in rat cortex would generate a permissive environment for transcription initiation from within *hsp70* coding region.

The influence of H4Ac in HSF1 DNA-binding affinity has been previously showed. Binding profiles of HSF1 to every HSE in the *Drosophila* genome revealed that H4Ac is a critical feature modulating HSF1 binding *in vivo* [24,50]. Moreover, disease progression in a genetic mouse model of Huntington, leads to decreased H4Ac in the brain, which correlates with a reduced ability of HSF1 to bind *hsp70* gene promoter despite its activation [51]. Thus, levels of H4Ac correlate with HSF1 binding to target elements.

Lower H3K4me2 in the *hsp70* promoter in cortical neurons compared with PC12 cells predicts a less prone binding site for transcription factors. Recent analysis of the ENCODE (Encyclopedia of DNA Elements) Consortium data-base revealed that H3K4me2 consistently defines TFBRs, with an overlapping score of ~90% between H3K4me2 and TFBRs in three different human cell lines. Likewise, regions with higher levels of H3K4me2 exhibit a higher overlapping percentage with TFBRs than regions with lower levels of H3K4me2 [44]. Thus, the data suggest that high H3K4me2 levels in the *hsp70* promoter in non-neuronal cells correlate with the strong potency of inducing Hsp70 during stress.

Expression of Hsp70 in the presence of TSA also indicates an association between H3 acetylation and efficient induction of Hsp70 expression. H3 acetylation was the only histone PTM significantly modified by heat shock in non-neuronal cells replicated in cortical neurons by TSA treatment, which associate with induction of Hsp70. The contribution of histone acetylation on Hsp70 expression induced by treatment with HDACs inhibitors in neurons is widely reported [22,23,28,52] and neuroprotective effects mediated by Hsp70 induction has been associated to Sp1 activation [23].

There is no doubt that HSF1 controls Hsp induction. HSF1 knockout mice present no induction of any Hsp in brain tissue [53]. However, the contribution of HSF1 to Hsp70 induced expression in neural tissue remains controversial. The possibility that HSF1 controls the heat shock response and survival in neurons in a non-canonical way is supported by a recent work. In this work by Verma et al, it was demonstrated that HSF1 effectively promotes neuroprotection through an Hsp-independent mechanism that does not require either HSF1 trimerization or the classical transactivation pathway [54].

Another interesting observation is that the induction of other Hsp proteins by heat shock, also dependent on the HSF1 activity, is not affected by neuronal differentiation. As we showed, no significant differences of Hsp25 mRNA induction were observed between differentiated and untreated PC12 cells or cortical neurons. These data strongly indicate that rather than something special preventing HSF1 action in neurons, the *hsp70* gene undergoes a modification during neuronal differentiation that prevents the action of HSF1 in this particular gene. Our studies on the transcriptomic profile of rat *hsp70* gene in the cerebral cortex, showing differential transcriptional start sites usage, support this idea. This fact added to particular differences on histone PTMs on *hsp70* gene promoter may also explain that distinct neuronal cell types induce different extents of Hsp70 [16,20,21,52,55].

In conclusion, we have confirmed that cells with neuronal phenotype exhibit a weaker induction of Hsp70 in response to stress. Our data indicate that this particular feature is owed mainly to the lack of binding of HSF1 to *hsp70* gene promoter determined by a less receptive chromatin landscape with a repressive contribution of HDACs.
